# Papilla and pontic area regeneration in patient with gingival smile: A clinical case

**DOI:** 10.4317/jced.54859

**Published:** 2018-05-01

**Authors:** Ramón Gómez-Meda, Carlos Torres-Sanchez, Santiago Mareque-Bueno, Juan Zufía-González, Daniel Torres-Lagares, José-Luis Gutierrez-Pérez

**Affiliations:** 1 DDS, MS, Private practice in Periodontics and prosthodontics, Ponferrada, León, Spain; 2 DDS, MS, PhD, Associate Research, Oral Surgery and Prosthodontics Department, Dentistry Faculty, University of Seville, Seville, Spain; 3 DDS MS, Private practice in Periodontics. Pontevedra, España. Profesor Santiago de Compostela University; 4 DDS, Private practice in Periodontics and prosthodontics, Madrid; 5 DDS, MS, PhD,Master Chief of Oral Surgery, Dentistry Department, University of Seville, Seville, Spain; 6 DMD, PhD, Master Chief of Oral Surgery, Dentistry Department, University of Seville, Seville, Spain

## Abstract

**Purpose:**

Connective tissue grafts are widely documented as a predictable technique for treating Miller Class I and II recessions, as well as procedures in which soft tissue augmentation is required for aesthetic reasons. This article aims to explore the resolution of a clinical case with this type of problema.

**Clinical case:**

This case describes a technique for reconstructing a pontic area and adjacent papilla by means of two consecutive connective tissue grafts. The first graft served to increase the amount of tissue in the horizontal direction, and the second promoted vertical reconstruction of the defect.

**Results and Conclusion:**

In cases with aesthetic requirements, restorative intervention may be able to mask tissue loss, but it can hardly achieve optimal aesthetic results. Periodontal plastic surgery techniques can be used to achieve that ideal result. The clinician must diagnose conditions in order to select correct treatment regimen for each individual case.

** Key words:**Papilla, gingival smile, pontic, restorative dentistry.

## Introduction

To achieve an aesthetic and harmonious smile, it is necessary to balance prosthetic restoration with gingival architecture and the patient’s lips and face. Several mucogingival techniques have been proposed for covering gingival recessions (1-4). The interdental papilla protects the periodontal structures, but it also plays an important role in aesthetics. Furthermore, its absence may lead to phonetic difficulties or food impaction (5). A lost dental papilla is difficult to regenerate. Very few cases have been identified in the literature (6-8), and there are no studies demonstrating a predictable technique for papilla reconstruction (9).

The success of rehabilitation therapy relies on more than just dental and functional restoration. Patients hope that prosthetic rehabilitation will create a harmonious, aesthetic smile with balanced mouth tissues. Therefore, dental aesthetics should be complemented with aesthetic soft tissues, which are an important topic of study nowadays (10).

Since Allen (11) described the foundations of soft tissue grafts for root coverage and their rational bases, these techniques have been used for covering implant recessions (12), as well as in the augmentation of soft tissues in aesthetic areas (6).

The use of these techniques as part of bone regeneration is justified when an increase in bone availability is needed for non-aesthetic reasons, as such aesthetic requirements can be properly achieved using soft tissue grafts (10).

The present case involves a surgical technique for the reconstruction of a pontic space with a bone defect and the adjacent missing papilla.

## Case Report

-Clinical and soft tissue parameters

**Figure 1:**
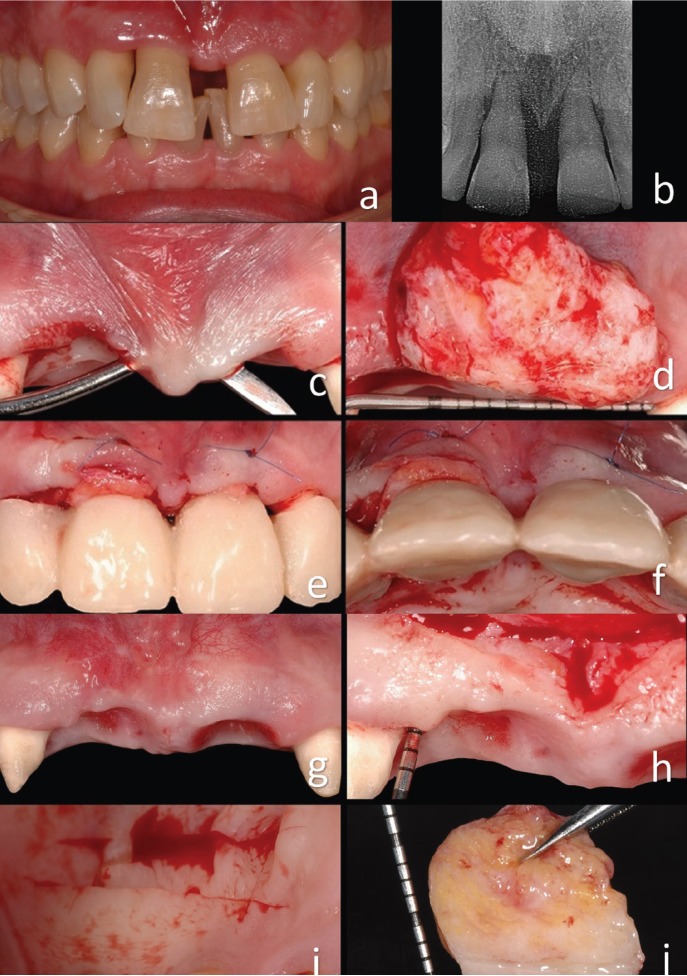
a) Initial situation of patient with a Miller Class III recession with loss of papilla between the right incisors. b) Bone support around central incisors. c, d, e, f) A first large connective tissue graft was positioned in the pontic area. g, h, i, j) After the first intervention, the tissue volume had increased, but it was clearly insufficient. Another connective tissue graft was carried out to compensate for this.

A 45-year-old non-smoker patient with periodontal defects around the upper incisors. The central incisors presented mobility type III and a probing depth of 10 mm and 8 mm circumferentially. The right lateral incisor also had a 10-mm probing depth at the mesial level, indicating bone loss under the papilla (Fig. 1a). It also had a 7-mm probing depth in the left lateral medial area. The patient’s medical history was not relevant: non-smoker, without allergies or chronic and/or repetitive pathologies. Some weeks prior to surgery, the patient was instructed in oral hygiene techniques and was subjected to another in-root scaling and root planing. At the time of diagnostic evaluation, the patient had a Miller Class III recession with loss of papilla between the right incisors; there was also a 2-mm recession in the vestibular area of both (Fig. 1a). Removal of the two central incisors was inevitable due to the surrounding attachment loss (Fig. 1b).

The patient’s smile showed gingival exposure, without harmony in the pink area. Photographic and radiographic records, impressions, and a facebow were all taken. The photographs were digitally analyzed, after which the models were mounted in an articulator and waxed following the digital analysis. The treatment alternatives and their determinants were presented and explained to the patient, and her choice was to undergo correction of the pontic area and try to correct the papilla defect through several connective tissue grafts. The tooth was not initially extracted for implant placement due to existing periodontal disease, which mobilizes teeth as a result of lack of support. Therefore, therapy with a fixed prosthesis was chosen to provide better retention of the final result of orthodontic and periodontal treatment. In this context, alveolar preservation (reduced by loss of support) is not necessary, as no implants were going to be placed. Under favorable conditions, a soft tissue graft is more predictable than a bone graft in obtaining proper results, and the former should therefore be chosen when bone augmentation is not necessary (10).

-Surgical procedure

Local anesthesia was administered using one carpule of articaine HC1 / epinephrine 40 / 0.005 mg / ml with epinephrine 1: 100,000 (Ultracain). The central incisors were extracted. The canines and lateral incisors were prepared, and a provisional fixed prosthesis was placed. Margin preparation of the lateral incisors was located supragingivally in mesial, given planned future reconstruction of the soft tissue. A canal root treatment was required in the lateral incisors. Silicone impressions were also taken to manufacture a second, more durable and precise temporary prosthesis.

The collapse of the soft tissues due to processes of vestibular reabsorption was very evident. A first large connective tissue graft was positioned in the pontic area (Fig. 1c-1f). The bases for the selection of this treatment option are described above. Incisions of partial thickness were made at the level of the concavities, preserving the papilla area tissue and consequently creating a tunnel between the two pontic areas. A 6/0 nylon suture was used to stabilize the graft without completely covering it. The temporary bridge did not place too much pressure on the graft (Fig.1c-1f) (1-4). Four months were allocated in order to achieve mature tissue. The tissue volume increased during this time but was still clearly insufficient (Fig. 1g-1j). More tissue was missing in the right central incisor and distal papilla area. The bone probing depth was 7 mm (Fig. 1g-1j); given a loss of 3 or 4 mm of papilla, there would therefore be a hypothetical probing depth of 10 mm and a 5-mm defect in the papilla area, which was rectified using another connective tissue graft.

**Figure 2:**
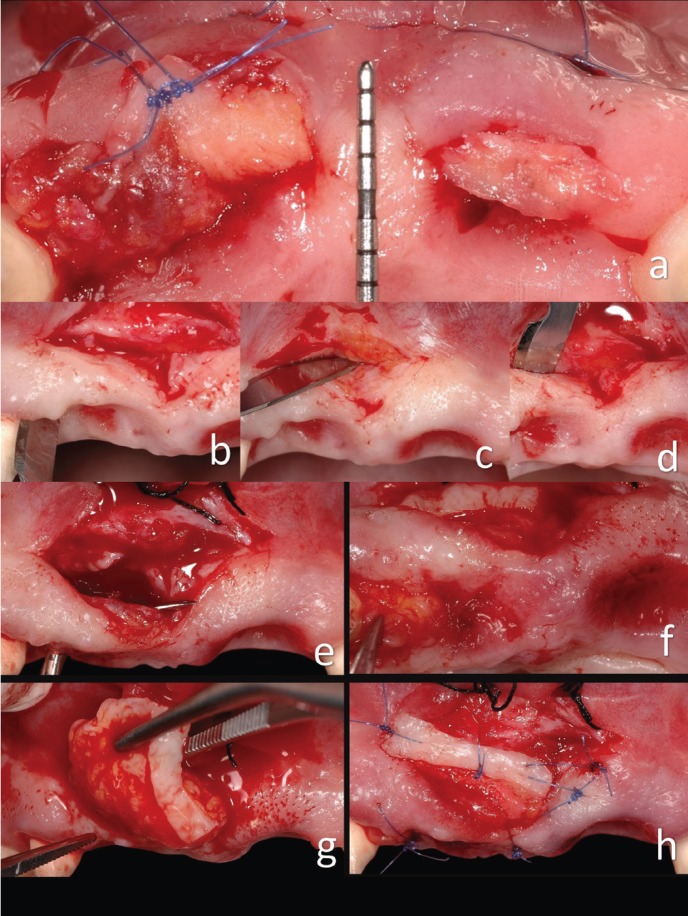
a) Situation after the first surgery. b, c, d) Intrasulcular incision in the second surgery. Incision over the frenulum. Incision making the vestibular tunel. e, f, g, h) Second connective tissue graft.

The second surgery was performed (Fig. 1g-1j). The initial preoperative situation was classified using Nordland and Tarnow’s table (9). The interdental papilla, vestibular gingiva, mucosa and palatine gingiva were anesthetized using Articaine HC1 / epinephrine 40 / 0.005 mg / ml with epinephrine 1: 100,000 (Ultracain) (8,9). A surgical dissection microscope was used to better visualize the surgical area. The first incision was of partial thickness with a semilunar shape, made from the mucogingival junction to reposition the large labial frenulum (Fig. 2a-2d) (3). The second incision was made using a microscalpel of the lost papilla around the neck of the lateral incisor. The blade was directed toward the bone to separate the connective tissue from the root surface. This incision enabled the preservation of the entire height and thickness of the gingival component, and it allowed access under the vestibular gingiva using miniature curettes. Surgical enlargement was used to preserve the integrity of the papilla (3,13).

The third incision was made at the apical edge with partial thickness in a semilunar shape and directed straight to the bone (Fig. 2e-2h). This incision released the gingival-papillary set. The mobility of this dento-papillary set was essential in enabling the creation of space under the papilla, necessary to receive a connective tissue graft. Mobility of palatal tissue was also achieved at the same time. The flap was positioned coronally using a curette under the groove and a small periosteotome under the pontic area (13). The amount of donor tissue required was determined using the initial gingival-incisal pre-surgical height, which was compared with the final desired papilla level (2,14).

A long, thick graft with a 2-mm layer of epithelium was removed from the palate (Fig. 1g-1j). This epithelial layer served to obtain denser fibrous connective tissue and to better maintain the space under the coronally positioned flap. The use of a large mass of connective tissue means that the graft’s chances of survival may increase due to greater surface area available for nutrition through blood perfusion. The epithelial layer was oriented towards the buccal groove of the coronally positioned flap, and it was not coated with the flap (Fig. 2e-2h) (11). The reason for this was that the epithelium is denser than the connective tissue and is therefore more adequate for supporting the repositioned flap. The connective tissue portion of the graft was placed in the furcation of the lost papilla to prevent the collapse of the flap and subsequent retraction of the papilla (Fig. 2e-2h). A continuous nylon 6/0 suture was used to stabilize the graft at the desired position, providing excellent wound stability. A microsurgical approach was taken using the Zeiss Omni Pico microscope. The wound in the palatal area was closed with a continuous suture (7,8).

**Figure 3:**
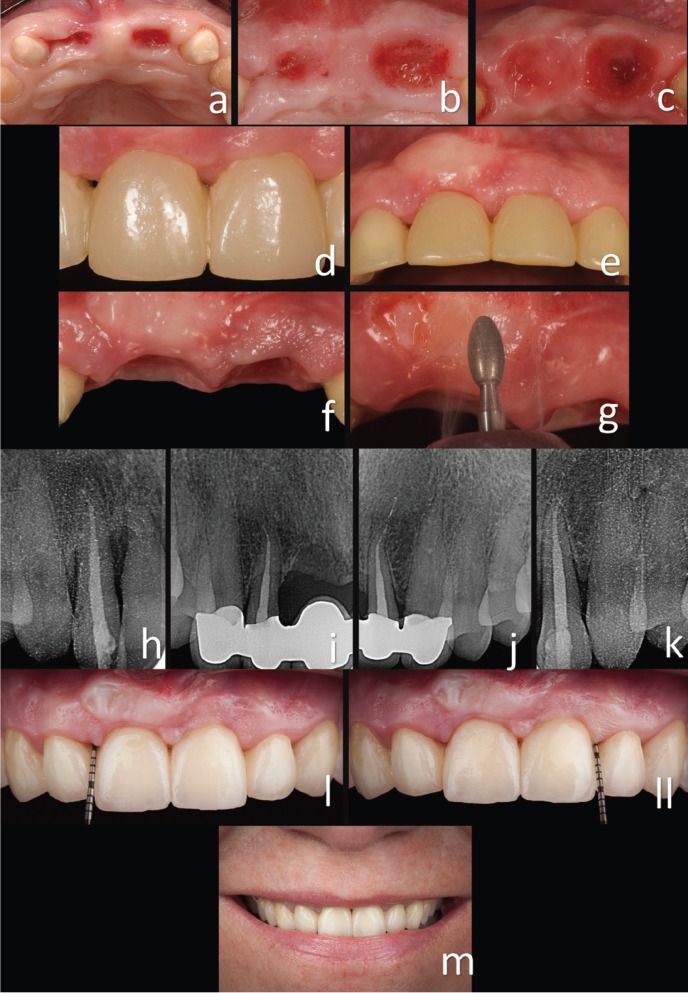
a, b, c) Improved soft tissue volume. d, e, f, g) The third surgical phase was performed before the final prostheses were placed. A diamond burn was used to remove part of the grafted epithelium. h, i, j, k) A significant improvement in the underlying bone was observed, despite no bone grafting having been carried out. l, ll) After the second surgery, the soft tissue margin of the papilla was 3–4 mm more incisal than before the surgery. This represented an improvement of approximately 4 mm in the periodontal junction. Three years later, the clinical results recorded within three months of surgery had not only been maintained but had in fact improved, as there was no “black triangle” in the lateral and central incisor area. m) Final situation after three years.

The patient was prescribed 500 mg of amoxicillin, to be taken every eight hours for ten days, and the patient was instructed to perform chlorhexidine rinses without alcohol twice a day for three weeks and to use a cotton bud soaked in chlorhexidine gluconate to remove any peeling epithelial cells or food debris in the intervened area (11). The sutures were removed four weeks after surgery as the patient lived far from the practice and could not travel before; under normal circumstances, the sutures could have been removed two weeks after surgery. The patient was told not to use any mechanical plaque control instruments in the intervened area for four weeks after surgery. Controls had previously been performed every week. The patient healed successfully and with no complications. The situation of the soft tissues at the level of the pontic area was very significantly improved (Fig. 3a-3c). The third surgical phase was performed before the final prostheses were placed. A diamond burn was used to remove part of the grafted epithelium (Fig. 3d-3g). The interdental area between the pontic area and the lateral incisors was not probed until six months later. A depth probe of 5 mm was recorded in the lateral incisor medial area, just 1-mm more than in the distal area, with no signs of swelling or bleeding. A significant improvement was observed in the underlying bone despite no bone grafting having been done (Fig. 3h-3k).

-Follow-up

The patient was evaluated three months after the first surgery. A horizontal increase had been achieved in the pontic area (Fig. 3h-3k). The probe depth was 7 mm in the lateral incisor mesial area before the second surgery. There was a recession of 3 mm and a Miller Class III recession in the right lateral incisor mesial area. After the second surgery, the papilla’s soft tissue margin was 3–4 mm more incisal than before the surgery (Fig. 3l-3ll). This represented an improvement in the periodontal junction of approximately 4 mm. Three years later, the clinical results recorded within three months of surgery had not only been maintained but had actually improved, as there was no “black triangle” in the lateral and central incisor area (Fig. 3h-3ll). There had been no contraction or retraction of the papilla, and the probe depth had not increased. Radiographic records showed that the underlying bone increased considerably (Fig. 3h-3k). The aesthetic aspect of the smile had also been substantially improved (Fig. 3m).

## Discussion

This case report showed a predictable soft tissue augmentation procedure in the tooth-supported fixed rehabilitation of an aesthetic area using a subepithelial connective tissue graft. Alternatives to subepithelial connective tissue grafts are supported by evidence of varying strength (15). Additional research is needed on the treatment outcomes for specific oral sites.

This procedure can be carried out using other periodontal plastic surgery techniques, and acellular dermal matrix grafts or enamel matrix derivatives may improve the obtained results when used in conjunction with these techniques (1). More clinical trials are necessary to assess the treatment outcomes for multiple-tooth recession defects, oral sites other than those involving maxillary canine and premolar teeth, and Miller Class III and IV defects (3,15).

While more clinical trials are needed, this case report showed that fixed prostheses using teeth as abutment remain a valid option for replacing lost dental pieces, especially as an alternative to complex vertical bone regeneration surgeries; these techniques require a greater number of follow-up appointments and therefore greater patient cooperation (13). The fixed prosthesis is thus a less risky option than implants when the patient lacks the necessary amount of soft and hard tissues. Although periodontal factors do not usually have a direct effect on the survival rate of a fixed prosthesis, harmony between the prosthesis and the periodontium is critical to aesthetics; otherwise, the longevity of the prosthesis and the periodontium will be compromised (3,4).

This case report showed that prosthetic design, the number and quality of the abutment teeth, preparation of the pontic area, the given occlusion, and the material used must all be considered when planning prosthodontic treatments (13). The location of the preparation margin and the contour and emergence profile of the prosthesis will influence the response of the gingival tissues to the prosthesis. Pontic design and cleansability also contribute to the response of the gingival tissues, as well as to their clinical and aesthetic outcome. Even an optimal pontic design will not prevent inflammation of the mucosa adjacent to the pontic area if pontic hygiene is not maintained by removing plaque. Case selection and the patients’ ability to maintain adequate oral hygiene are therefore essential to the longevity of prostheses, and regular follow-up appointments provide an opportunity for early detection and treatment of failures (5,9,13). Also important to consider are sociocultural and aesthetic expectations, as well as personal factors such as emotional resistance, which can reduce the risk of psychological trauma and possible failure.

Within the inherent limitations of this case report, it can be suggested that: 1) In cases with aesthetic requirements, restorative intervention can mask tissue loss, but it can hardly achieve optimal aesthetic results. Periodontal plastic surgery techniques can be used to achieve that ideal result. The clinician must diagnose all conditions in order to correctly select the best treatment for each individually case. 2) A close interdisciplinary relationship between periodontics and prosthodontics is therefore necessary to avoid unsatisfactory treatment outcomes that require extensive and expensive retreatment. 3) Surgical magnification and microsurgery instruments are advisable in order to give the clinician a better view of the area, avoid unnecessary incisions of discharge, and increase the predictability of the process.

## References

[1] Santamaria MP, Neves FLDS, Silveira CA, Mathias IF, Fernandes-Dias SB, Jardini MAN (2017). Connective tissue graft and tunnel or trapezoidal flap for the treatment of single maxillary gingival recessions: a randomized clinical trial. J Clin Periodontol.

[2] Cairo F, Pagliaro U, Buti J, Baccini M, Graziani F, Tonelli P (2016). Root coverage procedures improve patient aesthetics. A systematic review and Bayesian network meta-analysis. J Clin Periodontal.

[3] Azaripour A, Kissinger M, Farina VS, Van Noorden CJ, Gerhold-Ay A, Willershausen B (2016). Root coverage with connective tissue graft associated with coronally advanced flap or tunnel technique: a randomized, double-blind, mono-centre clinical trial. J Clin Periodontol.

[4] Gobbato L, Nart J, Bressan E, Mazzocco F, Paniz G, Lops D (2016). Patient morbidity and root coverage outcomes after the application of a subepithelial connective tissue graft in combination with a coronally advanced flap or via a tunneling technique: a randomized controlled clinical trial. Clin Oral Investig.

[5] Tarnow DP, Magner AW, Fletcher P (1992). The effect of the distance from the contact point to the crest of bone on the presence or absence of the interproximal dental papilla. J Periodontol.

[6] Ahmedbeyli C, Ipçi ŞD, Cakar G, Kuru BE, Yılmaz S (2014). Clinical evaluation of coronally advanced flap with or without acellular dermal matrix graft on complete defect coverage for the treatment of multiple gingival recessions with thin tissue biotype. J Clin Periodontol.

[7] Wang HL, Romanos GE, Geurs NC, Sullivan A, Suárez-López Del AF, Eber RM (2014). Comparison of two differently processed acellular dermal matrix products for root coverage procedures: a prospective, randomized multicenter study. J Periodontal.

[8] Pini Prato GP, Rotundo R, Cortellini P, Tinti C, Azzi R (2004). Interdental papilla management: a review and classification of the therapeutic approaches. Int J Periodontics Restorative Dent.

[9] Nordland WP, Tarnow DP (1998). A classification system for loss of papillary height. J Periodontol.

[10] Levine RA, Huynh-Ba G, Cochran DL (2014). Soft tissue augmentation procedures or mucogingival defects in esthetic sites. Int J Oral Maxillofac Implants.

[11] Allen AL (1994). Use of the supraperiosteal envelope in soft tissue grafting for root coverage. I. Rationale and technique. Int J Periodontics Restorative Dent.

[12] Hidaka T, Ueno D (2012). Mucosal dehiscence coverage for dental implant using split pouch technique: a two-stage approach. J Periodontal Implant Sci.

[13] Abduo J, Lyons KM (2017). Interdisciplinary interface between fixed prosthodontics and periodontics. Periodontol 2000.

[14] Graziani F, Gennai S, Roldán S, Discepoli N, Buti J, Madianos P (2014). Efficacy of periodontal plastic procedures in the treatment of multiple gingival recessions. J Clin Periodontol.

[15] Tatakis DN, Chambrone L, Allen EP, Langer B, McGuire MK, Richardson CR (2015). Periodontal soft tissue root coverage procedures: a consensus report from the AAP Regeneration Workshop. J Periodontol.

